# Limb-Shaking Transient Ischemic Attacks Successfully Treated with External Carotid Artery Stenting

**DOI:** 10.1155/2012/532329

**Published:** 2012-04-12

**Authors:** George N. Kouvelos, Christos Nassis, Nektario Papa, George Papadopoulos, Miltiadis I. Matsagkas

**Affiliations:** ^1^Vascular Surgery, Unit Department of Surgery, Medical School, University of Ioannina, 45110 Ioannina, Greece; ^2^Department of Intensive Care Unit, Medical School, University of Ioannina, 45110 Ioannina, Greece; ^3^Department of Anesthesiology, Medical School, University of Ioannina, 45110 Ioannina, Greece

## Abstract

The external carotid artery (ECA) is one of the most important extracranial-to-intracranial sources of collateral circulation, contributing significantly to the cerebral blood flow especially when perfusion through the internal carotid artery (ICA) is compromised. Most of the endovascular studies so far have been dedicated to ICA, with little focus on the ECA. Limb-shaking transient ischemic attacks (TIAs) are a relatively rare manifestation of carotid artery disease that may present with repetitive shaking movements of the affected limbs. We report a case of an 80-year-old male with bilateral internal and contralateral external carotid artery occlusion who developed limb-shaking TIAs as a result of significant stenosis of the right ECA. Percutaneous revascularization of the ECA was performed by angioplasty and stenting. At the follow-up 12 months later, the patient remained neurologically intact with complete resolution of his symptoms. Stenting of the ECA should be considered as a reasonable alternative to conventional open repair especially in patients with contralateral carotid stenosis, insufficient circle of Willis, and significant comorbidities.

## 1. Introduction

Transient ischemic attacks (TIAs) are usually brief neurological deficits resulting from focal cerebral ischemia not associated with permanent cerebral infarction [1]. They may present with symptoms such as loss of muscle power, sensation or vision. Hemodynamic failure is a relatively rare cause of TIAs. In these cases the patient may present with repetitive shaking movements of the affected limbs and is referred to as limb-shaking TIA [2]. This state may be triggered under conditions of reduced cerebral perfusion in patients with severe extracranial or intracranial occlusive disease [3].

We describe a case of limb-shaking TIAs in a patient with severe right external carotid artery (ECA) stenosis, bilateral internal carotid artery (ICA), and left ECA occlusion, in whom the development of limb-shaking TIAs was the manifestation of compromised cerebral perfusion. The patient was treated successfully by stenting of the right ECA.

## 2. Case Report

An 80-year-old male was referred to our department complaining of transient episodes of his left hand and foot shaking. These episodes would occur at 3-4 Hz lasting approximately for 1 min each with a frequency of 1 per week. During the episodes the patient was responsive with no significant alterations in his reactions. After the episode his clinical status normalized again. At the first assessment he was given a trial of anticonvulsants for a period of 3 months, but these did not reduce the frequency of the attacks. His past medical history was notable for coronary artery disease, hypertension, hyperlipidemia, and smoking.

**Figure 1:**
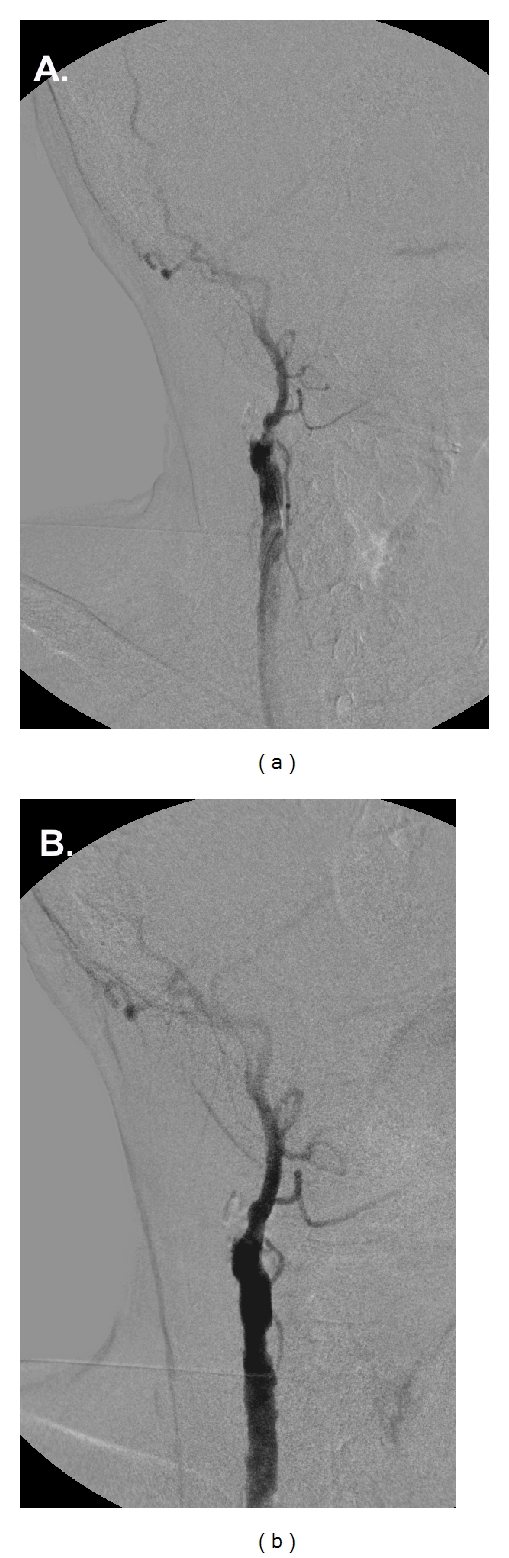
(a) Digital subtraction angiography showing the occluded right internal carotid artery and the significant stenosis of the right external carotid artery. (b) Completion angiogram after angioplasty and stenting of the right external carotid artery showing the resolution of the stenosis, as well as the significant enhancement on ECA's collateral circulation.

The patient proceeded with an MRI, which showed no evidence of acute stroke. An EEG depicted no evidence of epileptic activity. Color Doppler ultrasonography depicted complete occlusion of the right ICA, significant (85%) stenosis of the right ECA along with complete occlusion of the left ICA and ECA. Under local anesthesia and left common femoral artery access, a 0.014-inch guidewire was inserted percutaneously through an 8 F angled multipurpose catheter and passed through the external carotid lesion. Intravenous heparin was given at a dose of 100 IU/kg. A cerebral protection device (Accunet, Abbott Vascular, IL, USA) was navigated through the lesion and placed distal to the stenosis. A 6–8 × 40 mm tapered open-cell stent (Acculink, Abbott Vascular, IL, USA) was placed at the site of the occlusion and postdilated with a 5 × 20 mm balloon (Viatrac, Abbott Vascular, IL, USA). A completion angiogram confirmed the patency of external carotid artery with no residual stenosis (Figure 1).

The patient tolerated the procedure well with no neurologic sequel and was discharged home on the second postoperative day. At the follow-up appointments at the 1st, 6th, and 12th postoperative months, the patient remained neurologically intact with complete resolution of his symptoms. Color duplex ultrasonography showed patency of the ECA with no signs of restenosis.

## 3. Discussion

 The ipsilateral ECA can potentially provide an important collateral pathway for retinal and cerebral blood flow in the presence of occlusion or severe stenosis of the ICA, especially in patients with an incomplete circle of Willis [4]. In patients with symptomatic occlusion of the ICA, an ipsilateral stenosed ECA may precipitate additional sequel such as TIAs, stroke, and ocular disturbances [5]. These neurological symptoms can be due mainly to low perfusion or, less likely, to recurrent emboli carried to the brain through the collateral supply. In this setting, in the presence of ipsilateral ICA occlusion treating the ECA's stenosis could be an important therapeutic strategy in selected patients.

Limb-shaking TIAs are a rare form of TIAs that often become confused with focal motor seizures and therefore pose a diagnostic challenge. The diagnostic difficulty is compounded by the fact that cerebral ischemic damage is the most common cause of epilepsy in the elderly [2, 3]. Limb-shaking TIAs may represent an indicator of severe carotid occlusive disease, and these patients are at high risk of stroke. Limb-shaking TIA is a phenomenon associated to hypoperfusion at an ischemic territory linked to exhausted vasomotor reactivity [6]. Usually, the patient experiences transient rhythmic or arrhythmic involuntary movements and the face muscles are always spared, while EEG recording is normal. Treatment of limb-shaking TIAs has been reported in a series of case reports, but no controlled clinical trials have been conducted. Recently Turtzo et al. were the first to report on a patient with an ICA occlusion resolution of limb-shaking TIAs after angioplasty and stenting of the ECA [3]. On the other hand, Ribacoba et al. reported the only case with occlusive stenosis of the ICA without definite stroke presenting with partial motor status epilepticus [6]. Unfortunately, in this case although the patient was treated with ICA stenting, no data for the resolution of symptoms have been reported. To our knowledge, the present case is the second documentation of limb-shaking TIAs successfully treated with external carotid stenting.

Additionally, a branch of the ECA, the maxillary artery, supplies the masseter and temporalis muscles responsible for jaw movement and chewing. Occlusion of the ECA leading to symptomatic jaw claudication has been previously reported [7]. In our case, due to the occlusion of both ICAs and contralateral ECA, the preservation of the flow in the ipsilateral ECA was of the utmost importance, in order to preserve the flow of these important ECA branches and therefore preserve the normal supply to the facial muscles.

According to ECA treatment, carotid endarterectomy is the most common therapeutic strategy. Several studies have documented a low stroke rate and resolution or improvement of neurologic symptoms in patients after endarterectomy [8]. However, the long-term efficacy of ECA reconstruction in the prevention of stroke remains unknown. In our patient the temporary occlusion required for endarterectomy would have certainly exacerbated the cerebral ischemia, since there was also obstruction of the contralateral ICA and ECA. As it was demonstrated in the course of treatment, the additional collateral circulation provided by a patent ECA seemed enough to alleviate neurologic symptoms. Furthermore, in the case the symptoms had not resolved, stenting usually allows additional bypass options to be pursued in a second step. Although endarterectomy of ECA has been widely described in the literature, stenting has rarely been reported. Endovascular approach has been described for symptomatic stenosis, for augmentation of cerebral perfusion before extracranial to intracranial bypass and for treating carotid stump syndrome [9–11]. Xu et al. recently reported the largest series of ECA stenting concerning 12 patients [5]. In this series the authors suggest that ECA stenting seems to be safe and potentially effective strategy in the management of symptomatic ipsilateral ICA occlusion. In the present case a protection device for the prevention of embolic events during the procedure was used, as the atherosclerotic lesion of the carotid bifurcation was a significant source of emboli. In fact, many anastomotic pathways between the ECA and the intracranial cerebral circulation that are sufficiently dilated after concomitant ICA occlusion might have become routes for these emboli to pass into the brain. For these reasons, although randomized clinical trials are lacking, we believe that stenting of the ECA should be performed under distal brain protection.

In conclusion, endovascular stenting was successfully applied to treat a case of atherosclerotic ECA stenosis with bilateral ICA occlusion that presented with limb-shaking TIAs. Due to the small number of patients reported in the literature, no safe conclusions could be conducted concerning the safety of the procedure, the long-time effectiveness and the restenosis rate. Until larger studies will be reported, this modality should be considered as a reasonable alternative to conventional open repair especially in high-risk patients with contralateral carotid stenosis or occlusion and insufficient circle of Willis.
